# Supplementary material for: An Updated Systematic Review and Network Meta-Analysis of First-Line Triplet vs. Doublet Therapies for Metastatic Hormone-Sensitive Prostate Cancer
Source: Cancers (Basel). 2025 Jan 9;17(2):205. doi: 10.3390/cancers17020205 (PMC11763793; doi:10.3390/cancers17020205)
Supplement: Supplementary file 1 [file cancers-17-00205-s001.zip › cancers-3396979-supplementary.pdf]

Supplementary Information

Supplementary Table S1. PRISMA 2020 for Abstracts Checklist

Supplementary Table S2. PRISMA 2020

Supplementary Table S3. Summary of Network Meta-analyses for Each Regimen on Adverse Events

Supplementary Figure S1. Risk of Assessment using RoB2 tool
Supplementary Figure S2. Network Plot
Supplementary Figure S3. Network Meta-analysis for Radiographic Progression-free Survival
Supplementary Figure S4. Network Meta-analysis for Radiographic Progression-free Survival by Each Regimen

Supplementary File S1. Search strategy for systematic review and meta-analysis

Supplementary File S2. AMSTAR-2 checklist

Abbreviations:
AMSTAR-2 = A Measurement Tool to Assess Systematic Reviews 2, NMA = Network Meta-analysis, PRISMA = Preferred Reporting Items for Systematic Reviews and Meta-Analyses, RoB2 = Risk of Bias 2

Supplementary Table S1. PRISMA 2020 for Abstracts Checklist.

Section and Topic	Item #	Checklist item	Reported (Yes/No)
**TITLE**			
Title	1	Identify the report as a systematic review.	Yes
**BACKGROUND**			
Objectives	2	Provide an explicit statement of the main objective(s) or question(s) the review addresses.	Yes
**METHODS**			
Eligibility criteria	3	Specify the inclusion and exclusion criteria for the review.	Yes
Information sources	4	Specify the information sources (e.g. databases, registers) used to identify studies and the date when each was last searched.	Yes
Risk of bias	5	Specify the methods used to assess risk of bias in the included studies.	No
Synthesis of results	6	Specify the methods used to present and synthesise results.	Yes
**RESULTS**			
Included studies	7	Give the total number of included studies and participants and summarise relevant characteristics of studies.	Yes
Synthesis of results	8	Present results for main outcomes, preferably indicating the number of included studies and participants for each. If meta-analysis was done, report the summary estimate and confidence/credible interval. If comparing groups, indicate the direction of the effect (i.e. which group is favoured).	Yes
**DISCUSSION**			
Limitations of evidence	9	Provide a brief summary of the limitations of the evidence included in the review (e.g. study risk of bias, inconsistency and imprecision).	Yes
Interpretation	10	Provide a general interpretation of the results and important implications.	Yes
**OTHER**			
Funding	11	Specify the primary source of funding for the review.	No
Registration	12	Provide the register name and registration number.	Yes

Supplementary Table S2. PRISMA 2020.

Section and Topic	Item #	Checklist item	Location where item is reported
**TITLE**			
Title	1	Identify the report as a systematic review.	P1
**ABSTRACT**			
Abstract	2	See the PRISMA 2020 for Abstracts checklist.	P4-5
**INTRODUCTION**			
Rationale	3	Describe the rationale for the review in the context of existing knowledge.	P6
Objectives	4	Provide an explicit statement of the objective(s) or question(s) the review addresses.	P6
**METHODS**			
Eligibility criteria	5	Specify the inclusion and exclusion criteria for the review and how studies were grouped for the syntheses.	P7-9
Information sources	6	Specify all databases, registers, websites, organisations, reference lists and other sources searched or consulted to identify studies. Specify the date when each source was last searched or consulted.	P7-9
Search strategy	7	Present the full search strategies for all databases, registers and websites, including any filters and limits used.	P7-9
Selection process	8	Specify the methods used to decide whether a study met the inclusion criteria of the review, including how many reviewers screened each record and each report retrieved, whether they worked independently, and if applicable, details of automation tools used in the process.	P7-9
Data collection process	9	Specify the methods used to collect data from reports, including how many reviewers collected data from each report, whether they worked independently, any processes for obtaining or confirming data from study investigators, and if applicable, details of automation tools used in the process.	P7-9
Data items	10a	List and define all outcomes for which data were sought. Specify whether all results that were compatible with each outcome domain in each study were sought (e.g. for all measures, time points, analyses), and if not, the methods used to decide which results to collect.	P7-9
	10b	List and define all other variables for which data were sought (e.g. participant and intervention characteristics, funding sources). Describe any assumptions made about any missing or unclear information.	P7-9

Section and Topic	Item #	Checklist item	Location where item is reported
Study risk of bias assessment	11	Specify the methods used to assess risk of bias in the included studies, including details of the tool(s) used, how many reviewers assessed each study and whether they worked independently, and if applicable, details of automation tools used in the process.	P7-9
Effect measures	12	Specify for each outcome the effect measure(s) (e.g. risk ratio, mean difference) used in the synthesis or presentation of results.	P7-9
Synthesis methods	13a	Describe the processes used to decide which studies were eligible for each synthesis (e.g. tabulating the study intervention characteristics and comparing against the planned groups for each synthesis (item #5)).	P7-9
	13b	Describe any methods required to prepare the data for presentation or synthesis, such as handling of missing summary statistics, or data conversions.	P7-9
	13c	Describe any methods used to tabulate or visually display results of individual studies and syntheses.	P7-9
	13d	Describe any methods used to synthesize results and provide a rationale for the choice(s). If meta-analysis was performed, describe the model(s), method(s) to identify the presence and extent of statistical heterogeneity, and software package(s) used.	P7-9
	13e	Describe any methods used to explore possible causes of heterogeneity among study results (e.g. subgroup analysis, meta-regression).	P7-9
	13f	Describe any sensitivity analyses conducted to assess robustness of the synthesized results.	P7-9
Reporting bias assessment	14	Describe any methods used to assess risk of bias due to missing results in a synthesis (arising from reporting biases).	P7-9
Certainty assessment	15	Describe any methods used to assess certainty (or confidence) in the body of evidence for an outcome.	P7-9
**RESULTS**			
Study selection	16a	Describe the results of the search and selection process, from the number of records identified in the search to the number of studies included in the review, ideally using a flow diagram.	P10-13 Figure 1
	16b	Cite studies that might appear to meet the inclusion criteria, but which were excluded, and explain why they were excluded.	P10-13 Table1

Section and Topic	Item #	Checklist item	Location where item is reported
Study characteristics	17	Cite each included study and present its characteristics.	P10-13 Table1
Risk of bias in studies	18	Present assessments of risk of bias for each included study.	P10-13 Supplementary Information
Results of individual studies	19	For all outcomes, present, for each study: (a) summary statistics for each group (where appropriate) and (b) an effect estimate and its precision (e.g. confidence/credible interval), ideally using structured tables or plots.	P10-13 Table 1
Results of syntheses	20a	For each synthesis, briefly summarise the characteristics and risk of bias among contributing studies.	P10-13
	20b	Present results of all statistical syntheses conducted. If meta-analysis was done, present for each the summary estimate and its precision (e.g. confidence/credible interval) and measures of statistical heterogeneity. If comparing groups, describe the direction of the effect.	P10-13
	20c	Present results of all investigations of possible causes of heterogeneity among study results.	P10-13
	20d	Present results of all sensitivity analyses conducted to assess the robustness of the synthesized results.	NA
Reporting biases	21	Present assessments of risk of bias due to missing results (arising from reporting biases) for each synthesis assessed.	P10-13 Supplementary Information
Certainty of evidence	22	Present assessments of certainty (or confidence) in the body of evidence for each outcome assessed.	P10-13
**DISCUSSION**			
Discussion	23a	Provide a general interpretation of the results in the context of other evidence.	P14-17
	23b	Discuss any limitations of the evidence included in the review.	P14-17
	23c	Discuss any limitations of the review processes used.	P14-17
	23d	Discuss implications of the results for practice, policy, and future research.	P14-17
**OTHER INFORMATION**			

Section and Topic	Item #	Checklist item	Location where item is reported
Registration and protocol	24a	Provide registration information for the review, including register name and registration number, or state that the review was not registered.	Supplementary Information
	24b	Indicate where the review protocol can be accessed, or state that a protocol was not prepared.	P7
	24c	Describe and explain any amendments to information provided at registration or in the protocol.	NA
Support	25	Describe sources of financial or non-financial support for the review, and the role of the funders or sponsors in the review.	Authorship form
Competing interests	26	Declare any competing interests of review authors.	Authorship form
Availability of data, code and other materials	27	Report which of the following are publicly available and where they can be found: template data collection forms; data extracted from included studies; data used for all analyses; analytic code; any other materials used in the review.	NA

Supplementary Table S3. Summary of Network Meta-analyses for Each Regimen on Adverse Events.

	AEs		Hot Flush		Fatigue		Hypertension		Anemia		Neutropenia		FN	AST increase		ALT increase		ALP increase
	Any grade	Grade ≥3	Any grade	Grade ≥3	Any grade	Grade ≥3	Any grade	Grade ≥3	Any grade	Grade ≥3	Any grade	Grade ≥3		Any grade	Grade ≥3	Any grade	Grade ≥3	Any grade
**Summary of Included Studies**	8 studies	8 studies	5 studies	5 studies	7 studies	8 studies	5 studies	8 studies	6 studies	6 studies	3 studies	5 studies	3 studies	4 studies	6 studies	4 studies	6 studies	3 studies
	8 comparisons	8 comparisons	5 comparisons	5 comparisons	7 comparisons	8 comparisons	5 comparisons	8 comparisons	6 comparisons	6 comparisons	3 comparisons	5 comparisons	3 comparisons	4 comparisons	6 comparisons	4 comparisons	6 comparisons	3 comparisons
	7776 patients	7776 patients	4455 patients	4455 patients	6772 patients	6743 patients	5195 patients	7776 patients	5735 patients	5735 patients	2889 patients	4165 patients	2581 patients	3378 patients	5249 patients	3378 patients	5249 patients	2920 patients
**ADT**																		
**RR (95% CI)**	Ref.	Ref.	Ref.	Ref.	Ref.	Ref.	Ref.	Ref.	Ref.	Ref.	Ref.	Ref.	NA	Ref.	Ref.	Ref.	Ref.	Ref.
**P-score**	0.4329 (Rank 6)	0.70 (Rank 1)	0.72 (Rank 2)	0.59 (Rank 3)	0.72 (Rank 3)	0.64 (Rank 2)	0.82 (Rank 1)	0.51 (Rank 5)	0.57 (Rank 3)	0.39 (Rank 5)	0.81 (Rank 2)	0.90 (Rank 1)		0.69 (Rank 2)	0.80 (Rank 1)	0.66 (Rank 2)	0.74 (Rank 1)	0.25 (Rank 3)
**Abi+Doc+ADT**			NA	NA	NA		NA		NA		NA			NA		NA		NA
**RR (95% CI)**	0.92 (0.64-1.33)	1.53 (0.54-4.31)				2.40 (0.09-62.56)		0.30 (0.02-5.27)				35.33 (1.11-1134.00	0.96 (0.49-1.85)					
**P-score**	0.62 (Rank 2)	0.29 (Rank 8)				0.40 (Rank 5)		0.70 (Rank 2)				0.25 (Rank 5)	0.39 (Rank 2)					
**Dar+Doc+ADT**			NA	NA		NA	NA							NA		NA		NA
**RR (95% CI)**	0.93 (0.66-1.30)	1.31 (0.47-3.65)			3.75 (2.34-5.99)			0.35 (0.02-6.31)	3.61 (2.26-5.77)	1.84 (0.31-10.95)	18.74 (7.29-48.19)	32.77 (1.06-1014.25)	1.06 (0.70-1.59)		3.73 (0.50-27.98)		1.90 (0.14-26.24)	
**P-score**	0.61 (Rank 3)	0.43 (Rank 6)			0.08 (Rank 7)			0.63 (Rank 3)	0.03 (Rank 7)	0.25 (Rank 6)	0.15 (Rank 4)	0.27 (Rank 3)	0.26 (Rank 4)		0.28 (Rank 5)		0.50 (Rank 4)	
**Abi+ADT**																		
**RR (95% CI)**	1.00 (0.84-1.17)	1.32 (0.75-2.35)	1.22 (0.88-1.69)	0.50 (0.02-15.03)	0.94 (0.68-1.29)	1.25 (0.16-9.62)	1.74 (1.36-2.21)	2.10 (1.51-2.92)	0.69 (0.49-0.98)	0.63 (0.34-1.18)	0.91 (0.37-2.25)	1.61 (0.17-15.36)	0.05 (0.02-0.17)	1.36 (0.98-1.90)	3.26 (1.54-6.87)	1.32 (0.96-1.82)	6.12 (1.32-28.33)	0.29 (0.06-1.39)
**P-score**	0.434 (Rank 4)	0.38 (Rank 7)	0.37 (Rank 4)	0.68 (Rank 1)	0.77 (Rank 2)	0.56 (Rank 3)	0.11 (Rank 5)	0.10 (Rank 8)	0.90 (Rank 2)	0.73 (Rank 2)	0.86 (Rank 1)	0.82 (Rank 2)	1.00 (Rank 1)	0.34 (Rank 4)	0.32 (Rank 4)	0.31 (Rank 4)	0.16 (Rank 6)	0.89 (Rank 1)
**Apa+ADT**											NA	NA		NA		NA		
**RR (95% CI)**	1.00 (0.84-1.19)	1.03 (0.58-1.86)	1.39 (1.03-1.88)	1.01 (0.02-50.78)	1.18 (0.86-1.60)	1.34 (0.11-16.92)	1.14 (0.83-1.57)	0.92 (0.60-1.41)	0.68 (0,46-1.00)	0.53 (0.24-1.21)								0.57 (0.31-1.07)
**P-score**	0.430 (Rank 5)	0.62 (Rank 3)	0.19 (Rank 6)	0.54 (Rank 4)	0.50 (Rank 4)	0.52 (Rank 4)	0.62 (Rank 3)	0.56 (Rank 4)	0.91 (Rank 1)	0.81 (Rank 1)								0.70 (Rank 2)
**Dar+ADT**											NA	NA	NA					

**RR (95% CI)**	1.01 (0.80-1.28)	1.02 (0.54-1.92)	1.27 (0.70-2.32)	0.50 (0.01-25.17)	0.69 (0.37-1.29)	0.25 (0.00-15.01)	0.99 (0.56-1.76)	1.18 (0.51-2.74)	1.16 (0,77-1.74)	0.87 (0.36-2.10)				1.26 (0.70-2.25)	4.97 (0,63-39.04)	1.10 (0.62-1.97)	4.47 (0.35-57.49)	1.15 (0.59-2-24)
**P-score**	0.41 (Rank 8)	0.63 (Rank 2)	0.35 (Rank 5)	0.66 (Rank 2)	0.93 (Rank 1)	0.80 (Rank 1)	0.77 (Rank 2)	0.41 (Rank 6)	0.44 (Rank 5)	0.52 (Rank 4)				0.43 (Rank 3)	0.24 (Rank 6)	0.53 (Rank 3)	0.30 (Rank 5)	0.16 (Rank 4)
**Enz+ADT**											NA	NA	NA					NA
**RR (95% CI)**	1.00 (0.89-1.13)	1.18 (0.78-1.79)	1.22 (0.94-1.58)	4.02 (0.18-89.36)	1.26 (1.08-1.44)	2.69 (0.44-16.43)	1.64 (1.28-2.09)	1.89 (1.28-2.97)	1.04 (0.77-1.41)	0.77 (0.29-2.08)				0.41 (0.22-0.74)	0.99 (0.06-15.88)	0.70 (0.44-1.13)	1.32 (0.16-10.98)	
**P-score**	0.42 (Rank 7)	0.48 (Rank 4)	0.38 (Rank 3)	0.26 (Rank 6)	0.41 (Rank 5)	0.33 (Rank 6)	0.18 (Rank 4)	0.16 (Rank 7)	0.52 (Rank 4)	0.59 (Rank 3)				1.00 (Rank 1)	0.70 (Rank 2)	0.95 (Rank 1)	0.62 (Rank 3)	
**Doc+ADT**							NA											NA
**RR (95% CI)**	0.92 (0.68-1.25)	1.26 (0.54-2.95)	0.58 (0.41-0.84)	2.62 (0.69-10.05)	3.72 (2.46-5.74)	3.75 (0.41-31.03)		0.17 (0.01-3.01)	3.26 (2.18-4.89)	1.97 (0.36-10.88)	18.50 (7.36-46.52)	33.17 (2.56-429.33)	Ref	2.20 (1.20-4.04)	1.54 (.25-9.41)	1.92 (1.11-3.34)	1.17 (0.15-8.97)	
**P-score**	0.64 (Rank 1)	0.47 (Rank 5)	1.00 (Rank 1)	0.28 (Rank 5)	0.09 (Rank 6)	0.25 (Rank 7)		0.94 (Rank 1)	0.13 (Rank 6)	0.20 (Rank 7)	0.18 (Rank 3)	0.26 (Rank 4)	0.35 (Rank 3)	0.05 (Rank 5)	0.66 (Rank 3)	0.06 (Rank 5)	0.69 (Rank 2)	
**Cochran's Q test**	Q = 0.03; p = 0.9	Q = 6.59; p = 0.01	NA	NA	Q = 0.01; p = 0.9	Q = 10.23; p = 0.006	Q = 0.39; p = 0.53	Q = 0; p = 0.98	NA	NA	NA	Q = 2.45; p = 0.12	NA	NA	Q = 0.86; p = 0.35	NA	Q = 1.68; p = 0.19	NA

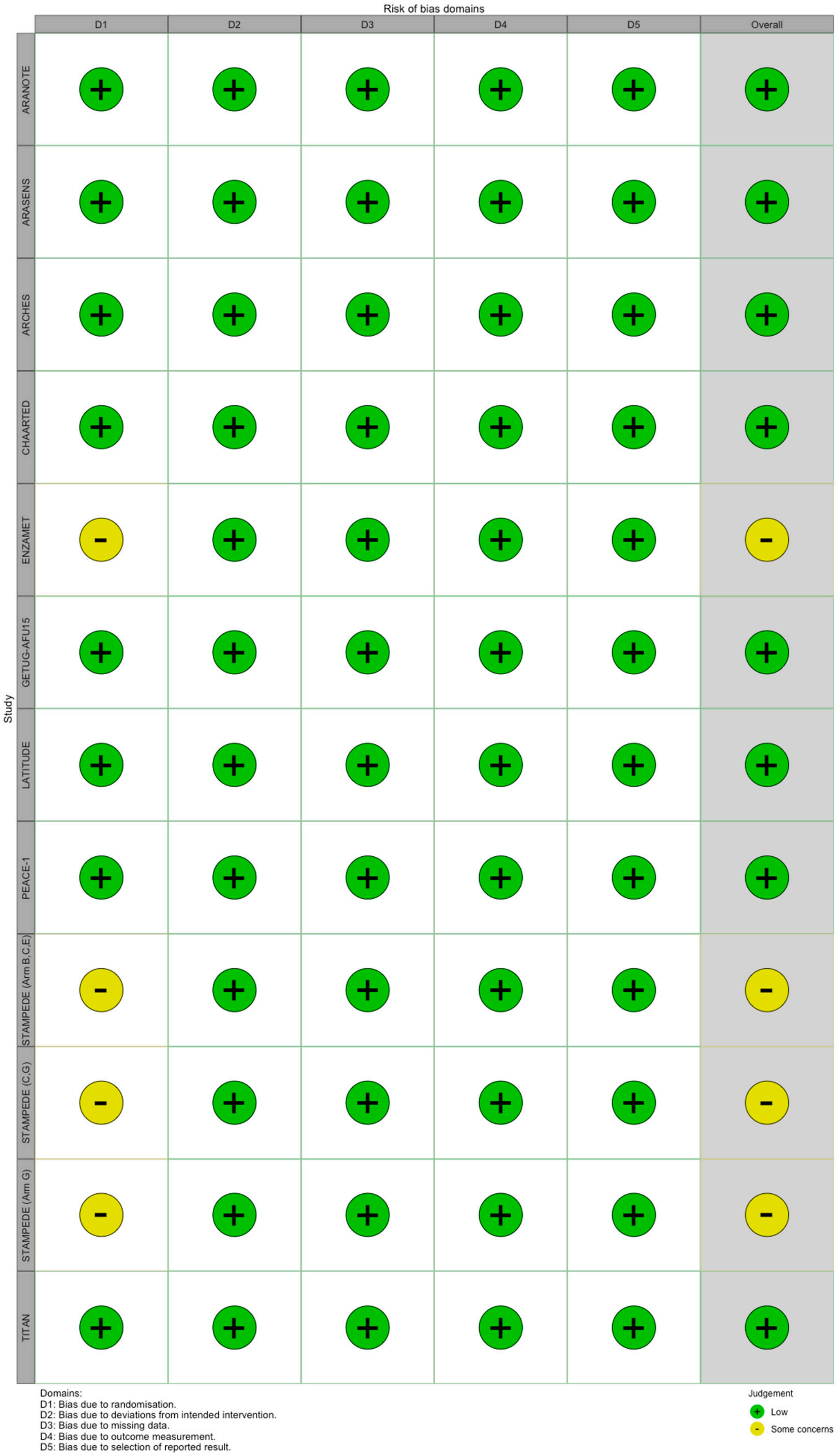

Supplementary Figure S1. Risk of Assessment using RoB2 tool.

(A) PFS

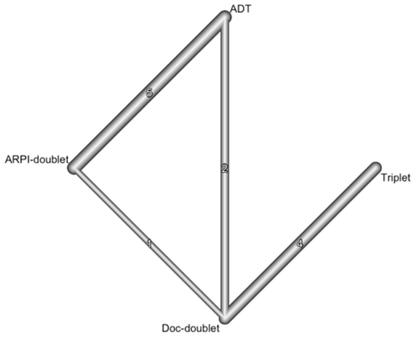

(B) PFS (Disease volume subgroup)

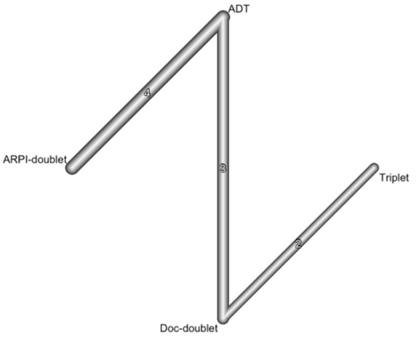

(C) OS

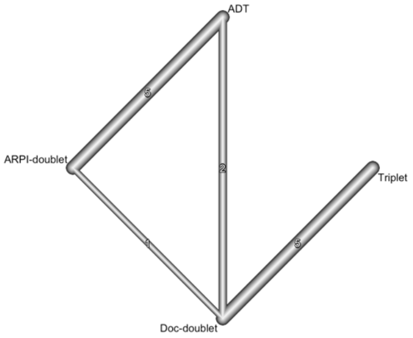

(D) OS (Disease volume subgroup)

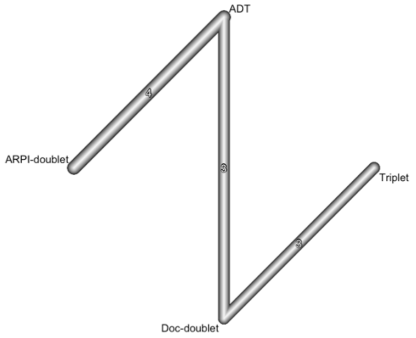

(E) Any grade AEs

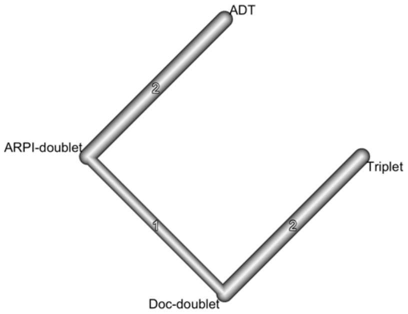

(F) Any grade AEs
(Disease volume subgroup)

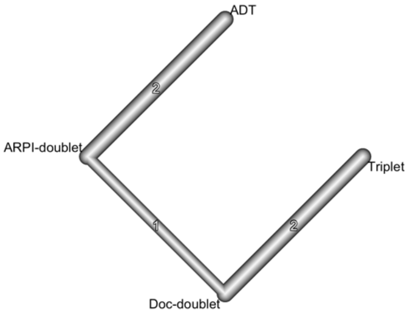

(G) PFS comparing of each regimen

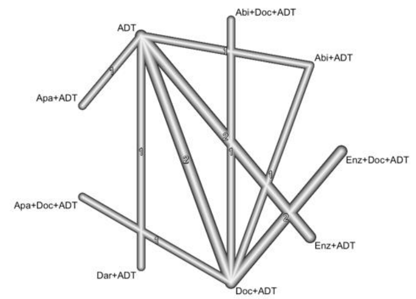

(H) PFS comparing of each regimen
(Disease volume subgroup)

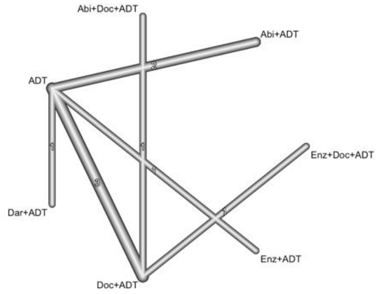

(I) OS comparing of each regimen

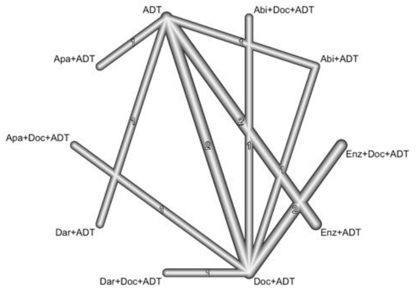

(J) OS comparing of each regimen
(Disease volume subgroup)

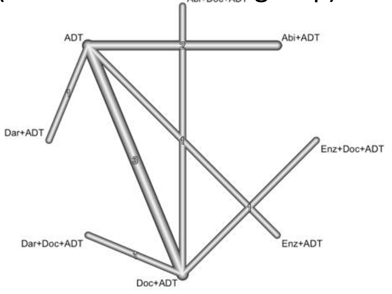

Supplementary Figure S2. Network Plot.

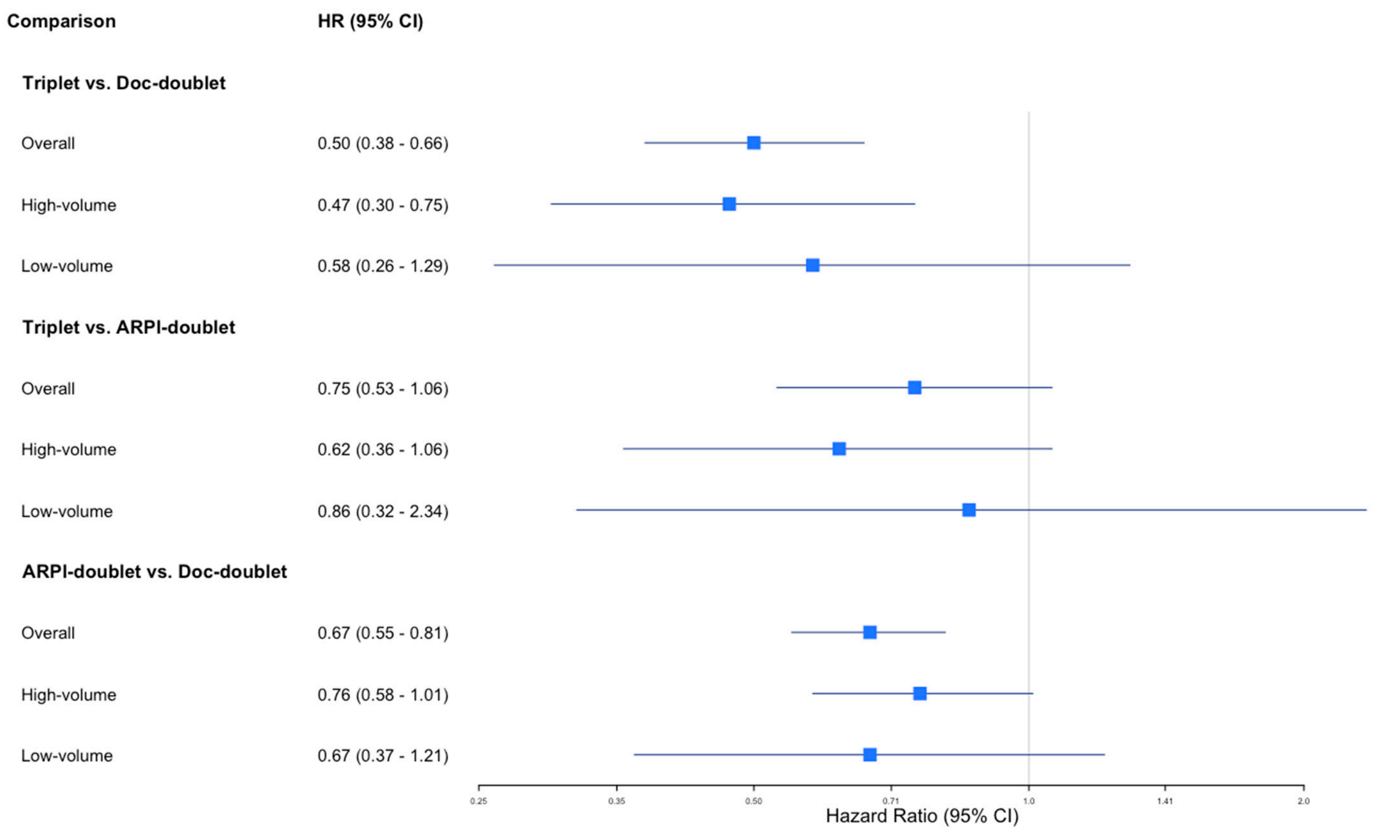

Supplementary Figure S3. Network Meta-analysis for Radiographic Progression-free Survival.

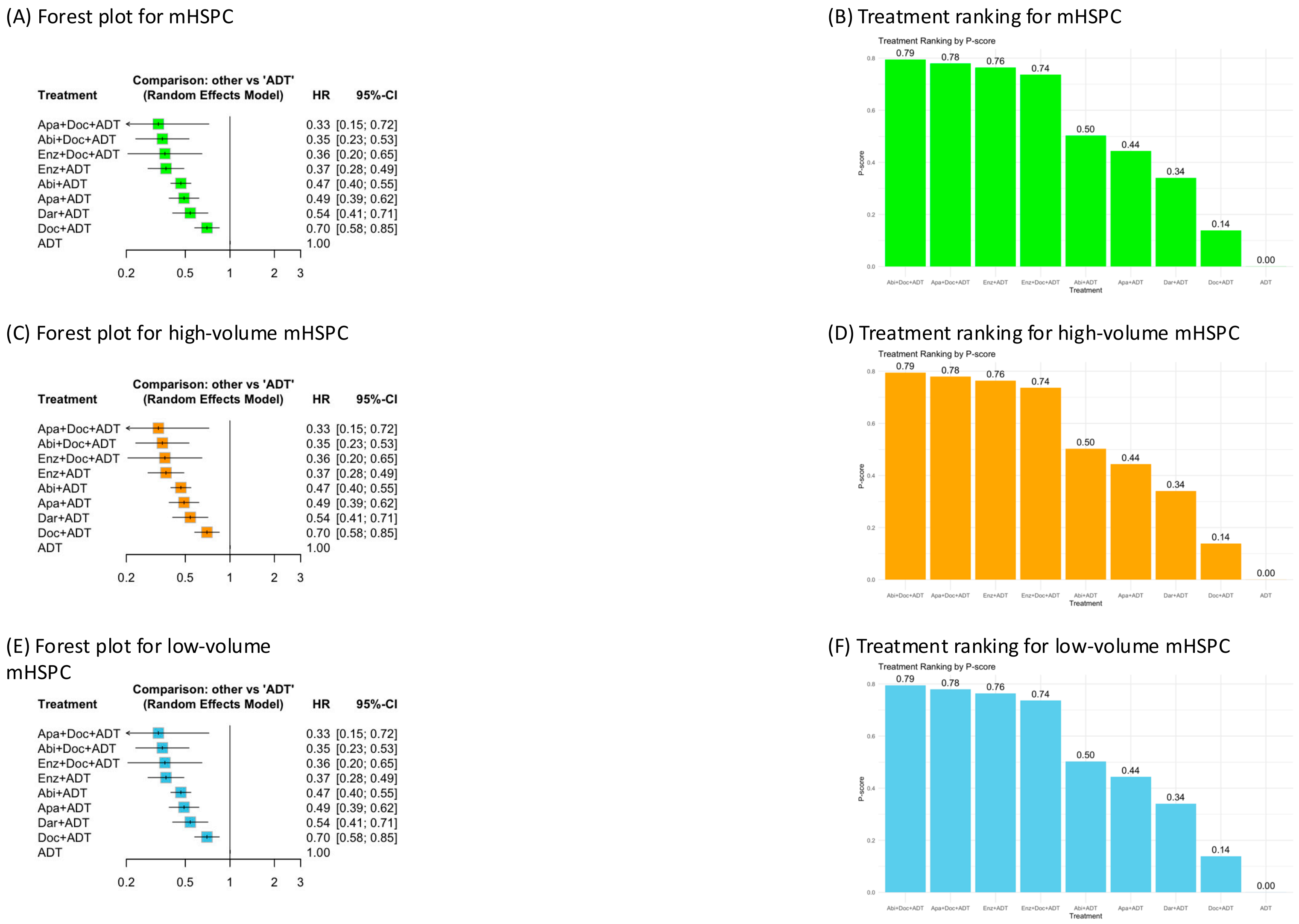

Supplementary Figure S4. Network Meta-analysis for Radiographic Progression-free Survival by Each Regimen.

Supplementary File S1. Search strategy for systematic review and meta-analysis.
Electronic searches were performed in the following databases to identify eligible studies

1. MEDLINE (via PubMed) (n = 279)
2. Embase (n = 633)
3. Web of Science (n = 464)

There were no language or publication period limitations.

MEDLINE (viaPubMed)
#1 Search: Prostate Neoplasms [MeSH Terms]
#2 Search: Prostate Cancer [Title/Abstract]
#3 Search: Noplasm Metastasis [MeSH Terms]
#4 Search: Metastatic [Title/Abstract]
#5 Search: Metastasis [Title/Abstract]
#6 Search: Advanced [Title/Abstract]
#7 Search: Neoplasms, Hormone Dependent [MeSH Terms]
#8 Search: Hormone-Dependent [Title/Abstract]
#9 Search: Hormone Sensitive [Title/Abstract]
#10 Search: Hormone Naive [Title/Abstract]
#11 Search: Castration Sensitive [Title/Abstract]
#12 Search: Castration Naive [Title/Abstract]
#13 Search: Progression Free Survival [Title/Abstract]
#14 Search: PFS [Title/Abstract]
#15 Search: Radiographic progression free survival [Title/Abstract]
#16 Search: rPFS [Title/Abstract]
#17 Search: Overall Survival [Title/Abstract]
#18 Search: OS [Title/Abstract]
#19 Search: Randomized Controlled Trials [Publication Type]
#20 Search: Randomized [Title/Abstract]
#21 Search: Randomised [Title/Abstract]
#22 Search: Randomly [Title/Abstract]
#23 Search: (#1 OR #2) AND (#3 OR #4 OR #5 OR #6) AND (#7 OR #8 OR #9 OR #10 OR #11 OR #12) AND (#13 OR #14 OR #15 OR #16 OR #17 OR #18 OR #19 OR #20 OR #21 OR #22)

Embase
'prostate cancer':ti,ab AND (metastatic:ti,ab OR metastasis:ti,ab OR advanced:ti,ab) AND ('hormone-dependent':ti,ab OR 'hormone sensitive':ti,ab OR 'hormone naive':ti,ab OR 'castration sensitive':ti,ab OR 'castration naive':ti,ab) AND ('progression free survival':ti,ab OR pfs:ti,ab OR 'radiographic progression free survival':ti,ab OR rpfs:ti,ab OR 'overall survival':ti,ab OR os:ti,ab) AND (randomized:ti,ab OR randomised:ti,ab OR randomly:ti,ab)

Web of Science

#1 TS = (Prostate Cancer)
#2 TS=(Metastatic)
#3 TS=(Metastasis)
#4 TS=(Advanced)
#5 TS=(Hormone Sensitive)
#6 TS=(Hormone Naive)
#7 TS=(Castration Sensitive)
#8 TS=(Castration Naive)
#9 TS=(Progression Free Survival)
#10 TS=(PFS)
#11 TS=(Radiographic Progression Free Survival)
#12 TS=(rPFS)
#13 TS=(Overall Survival)
#14 TS=(OS)
#15 TS=(Randomized)
#16 TS=(Randomised)
#17 TS=(Randomly)
#18 (#1) AND (#2 OR #3 OR #4) AND (#5 OR #6 OR #7 OR #8) AND (#9 OR #10 OR #11 OR #12 OR #13 OR #14) AND (#15 OR #16 OR #17)

Supplementary File S2. AMSTAR-2 checklist.

1. Did the research questions and inclusion criteria for the review include the components of PICO?

For Yes:
- [x] Population
- [x] Intervention
- [x] Comparator group
- [x] Outcome

Optional (recommended)
- [ ] Timeframe for follow up

- [x] Yes
- [ ] No

2. Did the report of the review contain an explicit statement that the review methods were established prior to the conduct of the review and did the report justify any significant deviations from the protocol?

For Partial Yes:
The authors state that they had a written protocol or guide that included ALL the following:
- [ ] review question(s)
- [ ] a search strategy
- [ ] inclusion/exclusion criteria
- [ ] a risk of bias assessment

For Yes:
As for partial yes, plus the protocol should be registered and should also have specified:
- [x] a meta-analysis/synthesis plan, if appropriate, and
- [x] a plan for investigating causes of heterogeneity
- [x] a plan for investigating causes of heterogeneity

- [x] Yes
- [ ] Partial Yes
- [ ] No

3. Did the review authors explain their selection of the study designs for inclusion in the review?

For Yes, the review should satisfy ONE of the following:
- [x] Explanation for including only RCTs
- [ ] OR Explanation for including only NRSI
- [ ] OR Explanation for including both RCTs and NRSI

- [x] Yes
- [ ] No

4. Did the review authors use a comprehensive literature search strategy?

For Partial Yes (all the following):
- [ ] searched at least 2 databases (relevant to research question)
- [ ] provided key word and/or search strategy
- [ ] justified publication restrictions (e.g. language)

For Yes, should also have (all the following):
- [x] searched the reference lists / bibliographies of included studies
- [x] searched trial/study registries
- [x] included/consulted content experts in the field
- [x] where relevant, searched for grey literature
- [x] conducted search within 24 months of completion of the review

- [x] Yes
- [ ] Partial Yes
- [ ] No

5. Did the review authors perform study selection in duplicate?

For Yes, either ONE of the following:
- [x] at least two reviewers independently agreed on selection of eligible studies and achieved consensus on which studies to include
- [ ] OR two reviewers selected a sample of eligible studies and achieved good agreement (at least 80 percent), with the remainder selected by one reviewer.

- [x] Yes
- [ ] No

6. Did the review authors perform data extraction in duplicate?

For Yes, either ONE of the following:
- [x] at least two reviewers achieved consensus on which data to extract from included studies
- [ ] OR two reviewers extracted data from a sample of eligible studies and achieved good agreement (at least 80 percent), with the remainder extracted by one reviewer.

- [x] Yes
- [ ] No

7. Did the review authors provide a list of excluded studies and justify the exclusions?

For Partial Yes:
- [ ] provided a list of all potentially relevant studies that were read in full-text form but excluded from the review

For Yes, must also have:
- [x] Justified the exclusion from the review of each potentially relevant study

- [x] Yes
- [ ] Partial Yes
- [ ] No

8. Did the review authors describe the included studies in adequate detail?

For Partial Yes (ALL the following):
- [ ] described populations
- [ ] described interventions
- [ ] described comparators
- [ ] described outcomes
- [ ] described research designs

For Yes, should also have ALL the following:
- [x] described population in detail
- [x] described intervention in detail (including doses where relevant)
- [x] described comparator in detail (including doses where relevant)
- [x] described study's setting
- [x] timeframe for follow-up

- [x] Yes
- [ ] Partial Yes
- [ ] No

9. Did the review authors use a satisfactory technique for assessing the risk of bias (RoB) in individual studies that were included in the review?

RCTs

For Partial Yes, must have assessed RoB from
- [ ] unconcealed allocation, and
- [ ] lack of blinding of patients and assessors when assessing outcomes (unnecessary for objective outcomes such as all-cause mortality)

For Yes, must also have assessed RoB from:
- [x] allocation sequence that was not truly random, and
- [x] selection of the reported result from among multiple measurements or analyses of a specified outcome

- [x] Yes
- [ ] Partial Yes
- [ ] No
- [ ] Includes only NRSI

NRSI

For Partial Yes, must have assessed RoB:
- [ ] from confounding, and
- [ ] from selection bias

For Yes, must also have assessed RoB:
- [ ] methods used to ascertain exposures and outcomes, and
- [ ] selection of the reported result from among multiple measurements or analyses of a specified outcome

- [ ] Yes
- [ ] Partial Yes
- [ ] No
- [ ] Includes only RCTs

10. Did the review authors report on the sources of funding for the studies included in the review?

For Yes
- [x] Must have reported on the sources of funding for individual studies included in the review. Note: Reporting that the reviewers looked for this information but it was not reported by study authors also qualifies

- [x] Yes
- [ ] No

11. If meta-analysis was performed did the review authors use appropriate methods for statistical combination of results?

RCTs

For Yes:

- [x] The authors justified combining the data in a meta-analysis
- [x] AND they used an appropriate weighted technique to combine study results and adjusted for heterogeneity if present.
- [x] AND investigated the causes of any heterogeneity

- [x] Yes
- [ ] No
- [ ] No meta-analysis conducted

For NRSI

For Yes:

- [ ] The authors justified combining the data in a meta-analysis
- [ ] AND they used an appropriate weighted technique to combine study results, adjusting for heterogeneity if present
- [ ] AND they statistically combined effect estimates from NRSI that were adjusted for confounding, rather than combining raw data, or justified combining raw data when adjusted effect estimates were not available
- [ ] AND they reported separate summary estimates for RCTs and NRSI separately when both were included in the review

- [ ] Yes
- [ ] No
- [ ] No meta-analysis conducted

12. If meta-analysis was performed, did the review authors assess the potential impact of RoB in individual studies on the results of the meta-analysis or other evidence synthesis?

For Yes:

- [x] included only low risk of bias RCTs
- [ ] OR, if the pooled estimate was based on RCTs and/or NRSI at variable RoB, the authors performed analyses to investigate possible impact of RoB on summary estimates of effect.

- [x] Yes
- [ ] No
- [ ] No meta-analysis conducted

13. Did the review authors account for RoB in individual studies when interpreting/ discussing the results of the review?

For Yes:

- [x] included only low risk of bias RCTs
- [ ] OR, if RCTs with moderate or high RoB, or NRSI were included the review provided a discussion of the likely impact of RoB on the results

- [x] Yes
- [ ] No

14. Did the review authors provide a satisfactory explanation for, and discussion of, any heterogeneity observed in the results of the review?

For Yes:

- [x] There was no significant heterogeneity in the results
- [ ] OR if heterogeneity was present the authors performed an investigation of sources of any heterogeneity in the results and discussed the impact of this on the results of the review

- [x] Yes
- [ ] No

15. If they performed quantitative synthesis did the review authors carry out an adequate investigation of publication bias (small study bias) and discuss its likely impact on the results of the review?

For Yes:

- [ ] performed graphical or statistical tests for publication bias and discussed the likelihood and magnitude of impact of publication bias

- [ ] Yes
- [ ] No
- [x] No meta-analysis conducted

16. Did the review authors report any potential sources of conflict of interest, including any funding they received for conducting the review?

For Yes:

- [ ] The authors reported no competing interests OR
- [x] The authors described their funding sources and how they managed potential conflicts of interest

- [x] Yes
- [ ] No
